# Rational and Design of the SIMULATOR Study: A Multicentre Randomized Study to Assess the Impact of SIMULation-bAsed Training on Transoesophageal echocardiOgraphy leaRning for Cardiology Residents

**DOI:** 10.3389/fcvm.2021.661355

**Published:** 2021-05-24

**Authors:** Théo Pezel, Anne Bernard, Yoan Lavie Badie, Julien Dreyfus, Etienne Audureau, Yohann Bohbot, Damien Fard, Arnaud Hubert, Lee S. Nguyen, Cécile Monteil, Loïc Bière, Florent Le Ven, Marjorie Canu, Sophie Ribeyrolles, Baptiste Mion, Basile Mouhat, Baptiste Bazire, Charles Fauvel, Julien Ternacle, Jennifer Cautela, Théo Cambet, Thierry Le Tourneau, Erwan Donal, Stéphane Lafitte, Nicolas Mansencal, Augustin Coisne

**Affiliations:** ^1^University of Paris, Department of Cardiology, Lariboisiere Hospital—APHP, Paris, France; ^2^INSERM UMRS 942, Paris, France; ^3^Ilumens Healthcare Simulation Department, Paris University, Paris, France; ^4^Division of Cardiology, Johns Hopkins University, Baltimore, MD, United States; ^5^Service de Cardiologie, CHRU de Tours, Toulouse, France; ^6^EA4245, Loire Valley Cardiovascular Collaboration, Université de Tours, Tours, France; ^7^Centre Régional d'Enseignement par la Simulation en Santé, Faculté de Médecine de Tours, Tours, France; ^8^Cardiac Imaging Center, Toulouse University Hospital, Toulouse, France; ^9^Cardiology Department, Centre Cardiologique du Nord, Saint-Denis, France; ^10^Clinical Epidemiology and Ageing (CEPIA), IMRB U955, UPEC, Creteil, France; ^11^CHU Henri Mondor, AP-HP, Creteil, France; ^12^Department of Cardiology, Amiens University Hospital, Amiens, France; ^13^UR UPJV 7517, Jules Verne University of Picardie, Amiens, France; ^14^Health Simulation Center SimUSanté®, Amiens University Hospital, Amiens, France; ^15^Department of Cardiology, Cardiology Intensive Care Unit, Henri-Mondor University Hospital, AP-HP, INSERM U955, Université Paris-Est Créteil, Créteil, France; ^16^Cardiologie, CHU de Rennes, LTSI, Rennes, France; ^17^Research and Innovation, RICAP, CMC Ambroise Paré, Neuilly-sur-Seine, France; ^18^Department of Cardiology, Angers University Hospital, Angers, France; ^19^Department of Cardiology, Brest University Hospital, CHRU de la Cavale Blanche, Brest, France; ^20^Department of Cardiology, Grenoble University Hospital, Grenoble, France; ^21^Department of Cardiology, Institut Mutualiste Montsouris, Paris, France; ^22^Department of Cardiology, University Hospital, Besançon, France; ^23^University of Paris, Department of Cardiology, Bichat Hospital—APHP, Paris, France; ^24^Department of Cardiology, CHU Rouen, FHU REMOD-VHF, Rouen, France; ^25^Hôpital Cardiologique Haut-Lévêque, CHU de Bordeaux, Pessac, France; ^26^Aix-Marseille University, University Mediterranean Center of Cardio-Oncology, Unit of Heart Failure and Valvular Heart Diseases, Department of Cardiology, North Hospital, Assistance Publique—Hôpitaux de Marseille, Centre for CardioVascular and Nutrition Research (C2VN), Marseille, France; ^27^Explorations fonctionnelles cardiovasculaires, Louis Pradel Hospital, Hospices Civils de Lyon, Bron, France; ^28^Inserm UMR1087, Institut du thorax, Université de Nantes, CHU de Nantes, Nantes, France; ^29^Department of Cardiology, Ambroise Paré Hospital, Assistance Publique-Hôpitaux de Paris (AP-HP), Centre de référence des cardiomyopathies et des troubles du rythme cardiaque héréditaires ou rares, Université de Versailles-Saint Quentin (UVSQ), Boulogne-Billancourt, France; ^30^INSERM U-1018, CESP, Epidémiologie clinique, UVSQ, Université de Paris Saclay, Villejuif, France; ^31^Department of Cardiovascular Explorations and Echocardiography—Heart Valve Clinic, CHU Lille, Lille, France; ^32^University of Lille, Inserm, CHU Lille, Institut Pasteur de Lille, U1011-EGID, Lille, France

**Keywords:** simulation-based, medical education, residents, transesophageal echoardiography, randomized study

## Abstract

**Introduction:** Simulation-based training in transesophageal echocardiography (TEE) seems promising. However, data are limited to non-randomized or single-center studies. To assess the impact of simulation-based vs. traditional teaching on TEE knowledge and performance for medical residents in cardiology.

**Materials and Methods:** Nationwide prospective randomized multicenter study involving 43 centers throughout France allowing for the inclusion of >70% of all French cardiology residents. All cardiology residents naive from TEE will be included. Randomization with stratification by center will allocate residents to either a control group receiving theoretical knowledge by e-learning only, or to an intervention group receiving two simulation-based training sessions on a TEE simulator in addition.

**Results:** All residents will undergo both a theoretical test (0–100 points) and a practical test on a TEE simulator (0–100 points) before and 3 months after the training. Satisfaction will be assessed by a 5-points Likert scale. The primary outcomes will be to compare the scores in the final theoretical and practical tests between the two groups, 3 months after the completion of the training.

**Conclusion:** Data regarding simulation-based learning in TEE are limited to non-randomized or single-center studies. The randomized multicenter SIMULATOR study will assess the impact of simulation-based vs. traditional teaching on TEE knowledge and performance for medical residents in cardiology, and whether such an educational program should be proposed in first line for TEE teaching.

## Introduction

Excellent know-how in transthoracic (TTE) and transesophageal echocardiography (TEE) is an essential requirement for the training of cardiology residents (1–3). Although TTE is rapidly taught and practiced on patients during the cardiology residency with dedicated studies (4), TEE remains too often neglected. The reference hands-on TEE teaching may be hampered by the availability of the teacher and equipment according to trainees' working patterns, and by the procedure, which is semi-invasive by itself with the need of esophageal intubation. Simulation may be a key educational tool to improve accessibility of TEE training and to accelerate the learning curve (5).

Despite a growing interest in simulation-based training (6, 7), its accessibility remains limited in the cardiology field. In a recent international survey performed with 172 young cardiologists from 43 countries, only 48% of the participants had already participated in a simulation training, while 91% considered this teaching method as “necessary” in cardiology (8). To respond to this training demand, several teams already offer to their residents a simulation education on TEE mannequin for several years (9). In France, simulation-based teaching in cardiology has also experienced significant developments, both technologically and institutionally. Indeed, simulation-based teaching is soon to become a legal obligation in the evaluation of graduate medical students (Health Law 2022) (10). However, as of yet, no national French simulation educational program exists in cardiology. The lack of studies evaluating simulation educational programs and the cost of TEE simulator may explain the lack of national consensus.

While supervised real-life TEE experience is essential, recent studies suggested that simulation-based TEE teaching is displaying significant benefit over conventional methods (11–17). To our knowledge, all these studies were non-randomized or randomized with limited single-centre sample size (11, 12, 14, 15, 18–23).

The SIMULATOR study is designed to assess the impact of SIMULation-bAsed Training on transoesophageal echocardiOgraphy leaRning for cardiology residents comparing simulation-based vs. traditional teaching on TEE knowledge and performance in cardiology residents, in several French centres. Our hypothesis is that skills and knowledge in TEE may be enhanced by the simulation-based TEE teaching.

## Methods

### Study Population and Centers

From November 2020 to November 2021, all consecutive residents in cardiology, of all training levels (year 1–4), who have never performed a TEE alone will be included in this randomized multicenter study conducted in 43 centers throughout France. The list of participating centers allowing for the inclusion of more than 70% of all French cardiology residents is provided in Supplementary Table 1. All residents who have already performed a TEE alone will be excluded. Residents will be invited via email from their local University coordinator to participate in the study.

The study will be approved by the Ethics committee of each center and authorized by the French data protection committee (*Commission Nationale Informatique et Liberte, CNIL*). Written informed consent will be obtained from all participants. Anonymized data supporting the findings of this study will be available from the corresponding author upon reasonable request. The study will be registered before starting the recruitment of the participants, and study data and results will be added at the completion of the study. All authors and future study investigators of this study have read and approved the manuscript as written.

### Randomization

Randomization with stratification by center will be performed at the individual (resident) level in 1:1 ratio, using a computer-based software (Research Randomizer 4.0; Social Psychology Network, Middletown, USA) to assign all the residents to the traditional group or to the simulation group. The random allocation sequence will be computer-generated by the statistician (E.A.) prior to the study using Stata v16.0 (StataCorp, College Station, TX, USA). Cluster-level randomization at the center level was not used for the present study, considering as minimal the risk of “contamination” of the effect of the training intervention in residents allocated in the control group.

### Study Design

**Figure 1:**
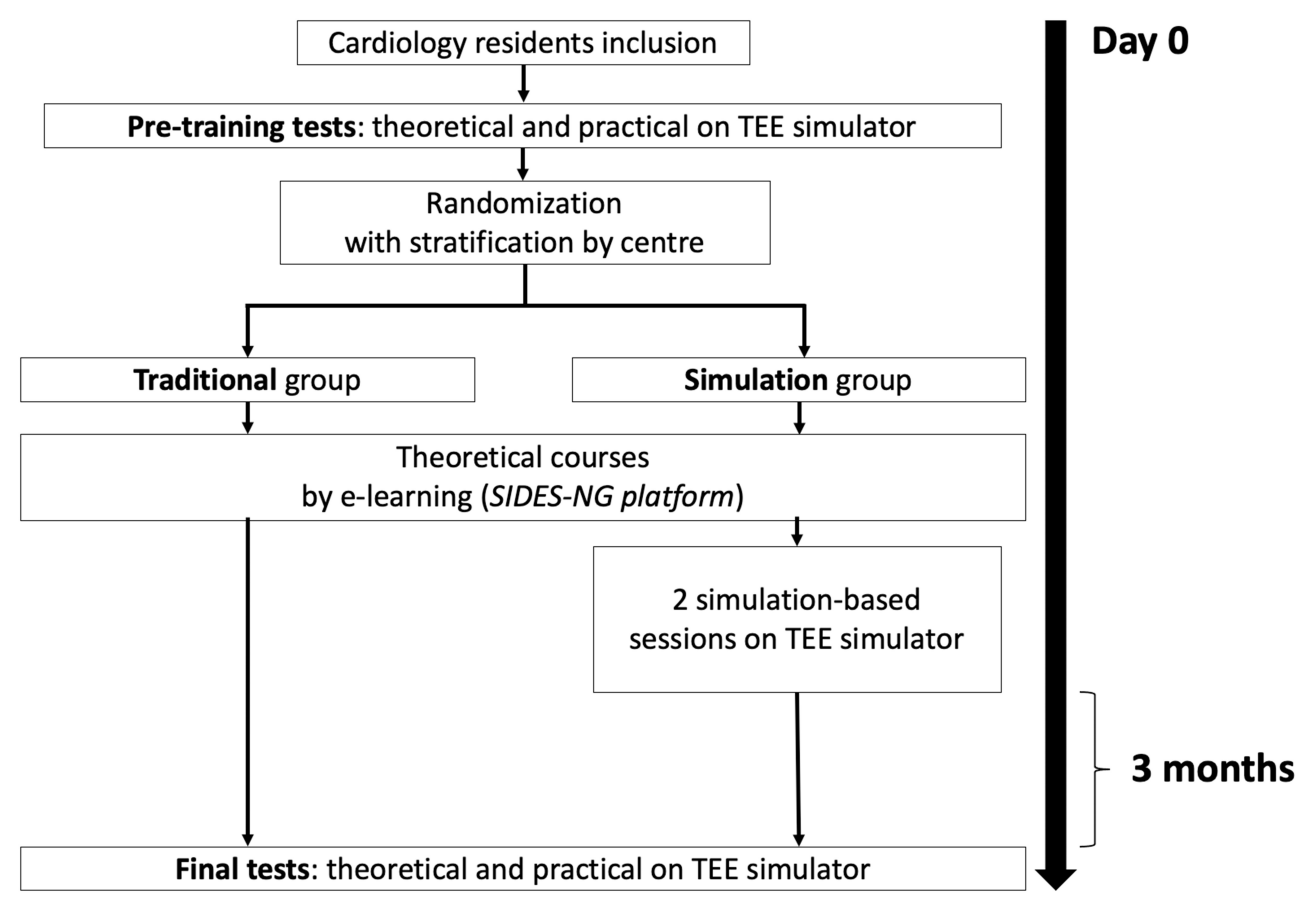
Flowchart of the study with schedule for training sessions and examinations in the two groups.

Each participant will complete two different tests during the study: (1) a pre-training test before starting the educational program; and (2) a final test performed 3 months after the end of the educational program. Each of these tests will include a theoretical test and a practical test on a TEE simulator (Figure 1). Notably, the pre-test will allow for an accurate assessment of each resident's theoretical and practical level in TEE at baseline.

### Baseline Participants Characteristics

The following demographic data will be collected: age, sex, residency semester, having the national diploma in echocardiography (validation on the SIDES platform), number of TTE performed, number of TEE observed, desire for a technical specialty in cardiology (cardiovascular imaging, interventional cardiology, electrophysiology, pediatric and congenital cardiology, or no technical specialty). All participants will answer a questionnaire on their prior experience of video games in childhood or currently, defined by more than 1 hour per week, as this seemed to be associated with performance on simulation-based models in previous studies (15, 24).

### Theoretical Test

The theoretical test will include 20 video-based questions online that evaluate recognition of standard TEE views, normal anatomy and some cases of mitral regurgitation, as already described (13, 19, 22, 23). Beyond the mitral regurgitation, the presence and severity of pathology and advanced TEE skills such as quantification and haemodynamic calculations will not be assessed. The residents will be given 90 s to choose the best answer out of a multiple-choice of 5 for each question (23). These tests will be designed by the experts from the French Group of Cardiovascular imaging of the French Society of Cardiology. Each question will be scored on 5 points (5 points if all the propositions correct and 0 points if at least one error), for a total of 100 points per test.

### Practical Tests

**Figure 2:**
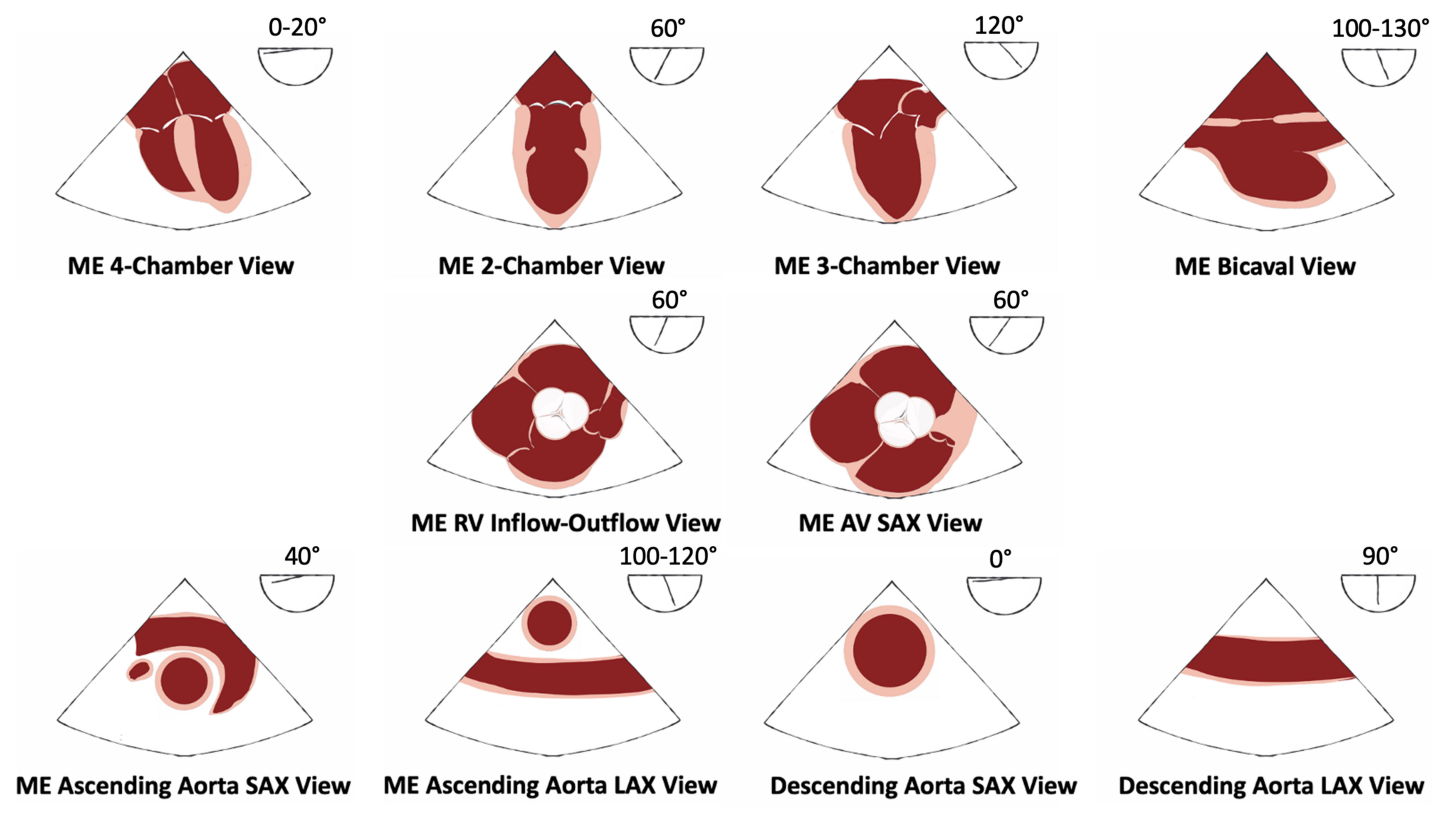
Ten basic TEE views for the practical test with anatomic structures assessed. AV, aortic valve; LAX, long axis; ME, midesophageal; RV, right ventricle; SAX, short axis; TEE, transesophageal echocardiography.

Immediately after each theoretical test (pre-training and final tests), all participants will be asked to demonstrate 10 basic views on a TEE simulator (Table 1), as previously described (14, 15). Of note, all residents of the two groups will have a few minutes before the practical test, to familiarize themselves with the handling of the simulator, without specific training and before the probe introduction. The standardized assessment that will be used by all teachers for the TEE grading scale of each views is presented in Figure 2. For each image, residents will have a maximum of 1 min to obtain their best view. The teacher will time the duration between TEE probe introduction and obtention of the specified view judged successful by the trainee. The modified Ferrero grading scale will be used to assess the quality of achievement of the views (14, 15, 23). In this grading system, each view will be marked out of 10 points according to the quality of the view. Of these 10 points, 5 will score for imaging angle and overall clarity of the view (poor quality = 0 points, average quality = 2 points, optimal quality = 5 points); and 5 will assess the presence of all the pertinent anatomic structures in the view (−1 point per missing structure not shown, and zero point if no structure identified) (Table 1). All teachers will ask for the 10 basic views in a pre-established order. Each view will be scored on 10 points, for a total of 100 points per practical test. These examinations will be evaluated by the local teacher, a certified national echocardiography teacher. Of note, residents will not be informed of their individual performance on theoretical or practical tests to avoid additional training beyond the study for performance purposes.

**Table 1:** List of basic TEE views, general anatomic structures (25, 26) and the mitral valve structures (27) that will be assessed in the study.

**Basic TEE views**	**General anatomic structures**	**Mitral valve structures**
ME 4-chamber	LA/LV/RA/RV/mitral valve	A2/P2
ME commissural view (2-chamber)	LV/Mitral valve/LA/LA appendage/circumflex artery/coronary sinus	P1/A2/P3 Antero-lateral commissure Antero-lateral muscle pillar
3-chamber	LA/LV/right coronary cusp/non-coronary cusp/mitral valve	A2/P2
ME bicaval	Interatrial septum/RA/LA/vena cava	
ME RV inflow-outflow	RV free wall/RV outflow tract/tricuspid valve	
ME aortic valve SAX	Right coronary, left coronary and non-coronary cusps/pulmonary valve/tricuspid valve/inter atrial septum	
Descending aorta SAX	Descending aorta/inferior wall/superior wall	
Descending aorta LAX	Descending aorta/round shape/wall (without interruption)	
ME ascending aorta SAX	Superior vena cava/ascending aorta/aortic valve/pulmonary artery	
ME ascending aorta LAX	Right pulmonary artery/ascending aorta/aortic valve	

*LA, left atrium; LAX, long axis; LV, left ventricle; ME, midesophageal; RA, right atrium; RV, right ventricle; SAX, short axis; TEE, transesophageal echocardiography*.

### Traditional and Simulation-Based Training

After the pre-training test, all participants will complete the following training sessions corresponding to the randomization. The first group (“traditional group”) will take part in a traditional didactic training using e-learning with the free-access online course *on the SIDES-NG platform* (website: https://www.uness.fr/plateformes-sides/sides-ng). This internet-based resource was developed by the French Group of Cardiovascular of the French Society of Cardiology and is freely accessible online. No session on TEE simulator will have to be performed during the study. The second group (“simulation group”) will access online courses too. Additionally, they will receive two one-on-one teaching sessions using a TEE simulator. The teaching program will be designed to facilitate sequential TEE examination according to ESC guidelines (3). The simulation session will involve standardized initial teaching of normal cardiac, including anatomy of mitral valve with some mitral regurgitation cases, aortic valve, tricuspid valve, interatrial septum, and left atrial appendage, and demonstration of image acquisition by the teacher (time duration: 30 min) (25). The duration of each session will be 2 h with a 6:1 instructor to student ratio. Each subject will have a dedicated 20 min of hands-on to manipulate the probe and undertake a sequential TEE examination under the supervision of the teacher. Instruction and feedback will be primarily verbal, but physical assistance with probe movement will also be provided to obtain and optimize the 10 basic views. Other participants can watch their colleagues working on the TEE simulator. The time allocated on TEE simulator will be divided between the acquisition of basic views and normal cardiac anatomy, including anatomy of mitral valve, aortic valve, tricuspid valve, interatrial septum, and left atrial appendage (first session), and an in-depth learning of mitral valve anatomy in TEE using some cases of mitral regurgitation (second session). The two practical sessions will be taught by the same instructor in each center. The maximum time between the two sessions will be 2 months.

Three months after completion of the study, all participants will be invited to a final test to assess long-term retention. Participants will be asked not to access additional learning resources about TEE between the pre- and final tests. All participants in both groups will be allowed to perform TEE during their daily clinical practice throughout the study. They will have to quantify prospectively the number of TEE observed or performed after training using a personal logbook provided by the teacher.

### TEE Simulator Protocol

For the “simulation group,” the same mannequin-based TEE simulators will be used for the study (“U/S Mentor Simulator,” 3D Systems-Simbionix, Littleton, USA). Functionalities of TEE simulators have been described previously (13). All trainers of the study are certified teachers in echocardiography and are accustomed to use simulation as a teaching method. To standardize the training and the practical test on TEE simulator, all trainers will follow a webinar of 30 min presenting the entire content of each session and the final test. Eighteen simulators will be used for this study. Some University centers already have their own simulator and will perform the study without additional cost. For the other centers which do not have a simulator, we will be able to set up a system of delivery of the simulator on the other centers during a limited period allowing to carry out the study.

### Satisfaction Assessment

After completion of the two training sessions, the participant satisfaction will be assessed by an anonymous questionnaire on the quality and effectiveness of the TEE training received during this study for the simulation group. As already published (15, 19), the questionnaire will include six statements on different aspects of the training such as (1) overall satisfaction and usefulness; (2) perceived benefit; (3) duration of the sessions; (4) relevance of the current level of training; (5) ideas of improvement; and (6) whether the participants would recommend this educational program for others. Satisfaction will be assessed using a 5-point Likert scale from 1 = strongly disagree to 5 = strongly agree. Responses will be deemed negative (Likert scale 1 and 2), neutral (Likert scale 3), or positive (Likert scale 4 and 5). The satisfaction of each participant will be scored on a total of 30 points.

### Outcomes

The coprimary outcomes of the study to compare the two groups will be the scores in the final theoretical and practical tests after the training will be completed.

The secondary outcomes will be the change in theoretical and practical tests scores from pre- to final-training. In addition, we will assess the satisfaction of participants.

### Statistical Analysis

#### Sample Size Calculation

Based on recent available literature (13, 15, 19, 21) and considering normalized 0–100 points score ranges for the two co-primary outcomes, a minimally important difference of 5 points (standard deviation 7 points) will be considered for the difference in change from pre- to post-training scores in theoretical and practical tests between the two randomized groups. Under these assumptions, a sample size of 50 subjects per group (for an overall population of 100 participants) will provide 90% power to detect a statistically significant difference between the two groups at a significance level of alpha = 2.5%, applying a Bonferroni correction to account for multiple testing of the two co-primary outcomes.

#### Statistical Methods

Demographic characteristics (age, sex, residency semester), having the national diploma in echocardiography, number of TTE performed, number of TEE observed, desire for a technical specialty in Cardiology collected at the time of randomization, will be summarized and compared between participants of the “traditional group” and the “simulation group.” Continuous data will be reported as means ± standard deviation (SD) for normally distributed data or as medians and interquartile range (IQR) for non-normally distributed data, as assessed through graphical methods and the use of the Shapiro-Wilk test for normality. Categorical data will be reported as counts and percentages. Between-groups comparisons will be performed using Student's *t*-test or Mann–Whitney test for continuous variables and using the Chi-2 or Fisher's exact test for categorical variables, as appropriate. Regarding the co-primary outcomes, on-parametric approaches (i.e., Mann-Whitney tests) will be favored over parametric tests due to the skewed distribution of test scores previously described in the literature (15). For within-groups comparisons (pre- vs. final tests), paired *t*-tests and Wilcoxon signed-rank tests will be performed for continuous variables. Longitudinal analysis of the evolution of the co-primary outcomes over the two time points (pre- and 3-month follow-up) will rely on mixed effects linear regression modeling, accounting for the correlation between repeated data over time. To address this risk of bias due the difference of number of TEE observed between the residents, an adjustment for the number of TEE observed will be performed in the final analysis. Prespecified subgroup analyses will be performed after stratifying by prior experience of video games in childhood or currently. A two-tailed *p* < 0.025 will be considered statistically significant for the primary analyses, and a two-tailed *p* < 0.05 for all other comparisons. All data will be analyzed using the *R* software, version 3.6.3 (R Project for Statistical Computing, R Foundation, Vienna, Austria) and Stata v16.0 (StataCorp, College Station, TX, USA).

## Discussion

This study will be the largest multicentre randomized study to assess the impact of simulation-based TEE teaching on the level of skills and practices of residents in cardiology. Simulation-based teaching in cardiology is experiencing a period of significant technological and institutional development. Further, simulation-based teaching has recently became a legal obligation in the evaluation of graduate medical students in France (10).

Previous studies evaluated the difference between TEE simulation training and conventional didactic training and e-learning (11, 12, 14, 20, 21, 23) and only three studies compared simulation training with hands-on training (15, 19, 22) considered as the “gold-standard” for TEE teaching. The majority of these studies showed superiority of TEE simulation training compared to traditional methods (12, 14, 15, 19–23). However, all of these studies were non-randomized (20) or randomized but limited by their sample size and monocentric character. Our multicentric randomized study with stratification by center will reduce the bias of variability in the quality and effectiveness of instructors, which is an essential step in the validation of an educational program. Besides, the majority of studies only evaluated the participants' retention in short-term recall 1 week after TEE education (19), and only a few studies performed long-term recall at 3 months (15). Only two small single-center studies have suggested a benefit of simulation training for residents who played video games with better visual, spatial and motor coordination than other students (15, 24). For that reason, we have chosen to also assess this element in the study.

Almost all of the studies evaluating the impact of simulation-based TEE teaching were performed with anesthesia residents (11, 13–15, 18–21, 23) or with cardiac surgery residents (22). To date, no study has been performed to assess the interest of simulation-based TEE teaching in cardiology residents. This may be explained by the earlier and faster development of simulation-based education in anesthesia-resuscitation and surgery compared with cardiology. However, TEE is most often challenging and stressful in cardiology as patients are usually awake under local anesthesia, and not under general anesthesia and as TEE exams usually need to provide a quick answer when guiding structural or surgical procedures with immediate consequences for the patient (26). Thus, TEE training in a safe environment with no risks for patients is particularly critical for cardiologists.

### Study Limitations

This study will have some limitations. First, residents of the traditional group will not manipulate any TEE probe during the training program. It could be argued that the opportunity for probe manipulation in the simulation group might confer an advantage in understanding anatomical relationships. Second, as previously described in the majority of educational studies (27). There is a risk of contamination of control residents, meaning the simulation group could discuss outside of the training sessions with the traditional group. However, the potential consequences of this risk are very limited, because the simulation-teaching experience is essentially practical.

Third, the practical test assessor will not be blinded due to the cluster randomization. Nevertheless, this assessment will be standardized for all assessors by the dedicated webinar.

Fourth, this study is an introduction to the practice of TEE and the use of simulators are not allowing for learning of probe insertion or the skills to manage the potential complications during *an* examination on real patients. Even though the simulation-based teaching should be even more beneficial for TEE with pathological cases which requires greater expertise. Indeed, the aim of our study was to evaluate the impact of simulation-based learning on basic TEE skills and knowledge. Further studies should assess its impact on more advanced level of expertise. Moreover, this study will not evaluate the educational contribution of simulated-based TEE training on actual patients. Indeed, this method is time-consuming and hardly compatible with clinical routine, with difficulty in finding patients with acceptable image quality for beginners. Hands-on TEE training may also present a patient safety issue, as TEE can potentially be traumatic for the patient, especially without general anesthesia. Finally, the financial aspect is another limitation of simulation-based training. Indeed, a significant and constant investment is necessary for the purchase of a simulator, for the maintenance and the update of the software. For this reason, simulation centers are most often based on multiple funding (University, national, regional and international grants, sponsorships).

## Conclusion

This multicentre randomized study will allow an assessment of the simulation-based TEE training impact compared to traditional teaching. Finally, the result of this study will highlight the interest of an educational program, including simulation-based TEE training.

## Data Availability Statement

The original contributions presented in the study are included in the article/Supplementary Material, further inquiries can be directed to the corresponding author/s.

## Ethics Statement

The studies involving human participants were reviewed and approved by Commission Nationale Informatique et Liberte (CNIL); Ethics committee of Lariboisiere hospital. The patients/participants provided their written informed consent to participate in this study.

## Author Contributions

TP, AC, AB, and EA designed the trial. TP and AC wrote the manuscript. EA performed statistical analyses. YB performed the illustration of the Figure 2. All the undersigning authors have substantially contributed to the paper. All authors reviewed the paper.

## Conflict of Interest

The authors declare that the research was conducted in the absence of any commercial or financial relationships that could be construed as a potential conflict of interest.
