# Supplementary material for: Rational and Design of the SIMULATOR Study: A Multicentre Randomized Study to Assess the Impact of SIMULation-bAsed Training on Transoesophageal echocardiOgraphy leaRning for Cardiology Residents
Source: Front Cardiovasc Med. 2021 May 24;8:661355. doi: 10.3389/fcvm.2021.661355 (PMC8180582; doi:10.3389/fcvm.2021.661355)
Supplement: Supplementary file 1 [file Table_1.DOCX]

**APPENDIX FILES**

**Supplementary Table 1. Detailed list of participating centers**

This table reports all participating centers with the respective number of residents officially available.

| Centers | Residents available (n) |
| --- | --- |
| Amiens-Picardie hospital (CHU), Amiens | 15 |
| Angers hospital (CHU), Angers | 10 |
| Assistance Publique - Hôpitaux de Marseille, Marseille | 15 |
| Besançon hospital (CHU), Besançon | 15 |
| Bordeaux hospital (CHU), Bordeaux | 30 |
| Brest hospital (CHU), Brest | 10 |
| Grenoble hospital (CHU), Grenoble | 15 |
| Lille University Hospital (CHU), Lille | 50 |
| Louis Pradel Hospital, Hospices Civils de Lyon, Bron | 30 |
| Nantes hospital (CHU), Nantes | 10 |
| Rennes hospital (CHU), Rennes | 25 |
| Rouen hospital (CHU), Rouen | 10 |
| Toulouse hospital (CHU), Toulouse | 30 |
| Tours (CHU), Tours | 20 |
| Poitiers (CHU), Poitiers | 15 |
| Ile de France, Ilumens centre (University of Paris), Paris   - Ambroise-Pare hospital (CHU), AP-HP, Boulogne - Andre-Mignot hospital, Versailles - Antoine-Beclere hospital, AP-HP, Clamart - Avicenne hospital (CHU), AP-HP, Bobigny - Bicetre hospital (CHU), AP-HP, Kremblin-bicetre - Bichat-Claude-Bernard hospital (CHU), AP-HP, Paris - Centre Cardiologique du Nord (CCN), Saint Denis - Centre Chirurgical Marie-Lannelongue hospital, Plessis - Clinique medico-chirurgical Ambroise Paré, Neuilly - Cochin hospital (CHU), AP-HP, Paris - HEGP hospital (CHU), AP-HP, Paris - Henri-Mondor hospital (CHU), AP-HP, Creteil - HIA Percy hospital, Clamart - Institut Mutualiste Montsouris, Paris - Lariboisiere- Fernand- Widal hospital (CHU), AP-HP, Paris - Le Raincy-Montfermeil hospital, Montfermeil - Marne La Vallee hospital, Marne La Vallee - Montreuil hospital, Montreuil - Necker Enfants Malades hospital (CHU), AP-HP, Paris - Pitie-Salpetriere hospital (CHU), AP-HP, Paris - Prive Jacques-Cartier hospital, Massy - Robert-Ballanger hospital, Aulnay - Robert-Debre hospital (CHU), AP-HP, Paris - Saint Antoine hospital (CHU), AP-HP, Paris - Saint Joseph hospital, Paris - Simone Veil hospital, Eaubonne - Sud-Francilien hospital, Corbeil - Victor-Dupouy hospital, Argenteuil - Villeneuve-St-Georges hospital, Villeneuve-St-Georges | 100 |
|  |  |
| Total potential inclusions | **385** |

*Abbreviations: AP-HP: Assistance publique – Hôpitaux de Paris, CHU: Centre Hospitalier Universitaire (University Hospital Centre).*
